# The Pollutant Organotins Leads to Respiratory Disease by Inflammation: A Mini-Review

**DOI:** 10.3389/fendo.2017.00369

**Published:** 2018-01-22

**Authors:** Albená Nunes-Silva, Dalton Dittz, Higor Scardini Santana, Rodrigo Alves Faria, Katia Michelle Freitas, Christiane Rabelo Coutinho, Livia Carla de Melo Rodrigues, Leandro Miranda-Alves, Ian Victor Silva, Jones Bernardes Graceli, Leandro Ceotto Freitas Lima

**Affiliations:** ^1^Department of Physical Education and Sports, Centro Desportivo da Universidade Federal de Ouro Preto, Ouro Preto, Brazil; ^2^Department of Pharmacology, Biological Sciences Institute, Universidade Federal de Minas Gerais, Belo Horizonte, Brazil; ^3^Department of Morphology, Universidade Federal do Espírito Santo, Vitória, Brazil; ^4^Department of Health Sciences, Universidade Federal do Espírito Santo, São Mateus, Brazil; ^5^Department of Pharmaceutical Sciences, Universidade Federal de Ouro Preto, Ouro Preto, Brazil; ^6^Cooperativa de Consumo dos Servidores do DER/MG (COOPEDER), Belo Horizonte, Brazil; ^7^Department of Physiological Sciences, Universidade Federal do Espírito Santo, Vitória, Brazil; ^8^Physiological Sciences Graduate Program, Universidade Federal do Espírito Santo, Vitória, Brazil; ^9^Research Group, Development in Experimental Endocrinology, Biomedical Science Institute, Universidade Federal do Rio de Janeiro, Rio de Janeiro, Brazil; ^10^Graduate Program in Endocrinology, Medicine School, Universidade Federal do Rio de Janeiro, Rio de Janeiro, Brazil; ^11^Graduate Program in Pharmacology and Medicinal Chemistry, Universidade Federal do Rio de Janeiro, Rio de Janeiro, Brazil

**Keywords:** airway disease, endocrine-disrupting chemicals, inflammation, organotin compounds, reactive oxygen species

## Abstract

Organotins (OTs) are organometallic pollutants. The OTs are organometallic pollutants that are used in many industrial, agricultural, and domestic products, and it works as powerful biocidal compound against large types of microorganisms such as fungi and bacteria. In addition, OTs are well known to be endocrine-disrupting chemicals, leading abnormalities an “imposex” phenomenon in the female mollusks. There are some studies showing that OTs’ exposure is responsible for neural, endocrine, and reproductive dysfunctions *in vitro* and *in vivo* models. However, OTs’ effects over the mammalian immune system are poorly understood, particularly in respiratory diseases. The immune system, as well as their cellular components, performs a pivotal role in the control of the several physiologic functions, and in the maintenance and recovery of homeostasis. Thus, it is becoming important to better understand the association between environmental contaminants, as OTs, and the physiological function of immune system. There are no many scientific works studying the relationship between OTs and respiratory disease, especially about immune system activation. Herein, we reported studies in animal, humans, and *in vitro* models. We searched studies in PUBMED, LILACS, and Scielo platforms. Studies have reported that OTs exposure was able to suppress T helper 1 (Th1) and exacerbate T helper 2 (Th2) response in the immune system. In addition, OTs’ contact could elevate in the airway inflammatory response, throughout a mechanism associated with the apoptosis of T-regulatory cells and increased oxidative stress response. In addition, OTs induce macrophage recruitment to the tissue, leading to the increased necrosis, which stimulates an inflammatory cytokines secretion exacerbating the local inflammation and tissue function loss. Thus, the main intention of this mini-review is to up to date the main findings involving the inflammatory profile (especially Th1 and Th2 response) in the respiratory tract as a result of OTs’ exposure.

## Introduction

The high levels of toxic compounds in the biosphere are elevating the human needs of better comprehension on their impact on earth life and modern society. The utilization of agricultural chemicals products is raising in the world. Concomitantly, the scientific community can observe the incidence of many diseases, such as diabetes, neurodegenerative diseases, asthma, and fertility alterations (1). According to World Health Organization, some toxic compounds are capable of inducing alterations in the endocrine system *via* inflammatory commitment and have been named as endocrine-disrupting chemicals (EDCs). Nowadays, is well known that EDCs is able to modulate important hormone-signaling pathways activities and have received great attention as an inductor of reproductive abnormality and also a chronic obstructive pulmonary disease (2).

Organotins (OTs) is well known as a EDCs. The OTs are organometallic pollutants used in various domestic, industrial, and agricultural products, as a powerful biocidal compound that works against large types of microorganisms, such as fungi and bacteria (3). Summarizing, OTs are frequently used as a chemical part of commercial products because they have a strong biocidal activity against a large spectrum of microorganisms as mentioned before. Tributyltin (TBT), that is a class of OTs, contains (C4H9)3Sn group and are used in many industry products: an example, in wood preservation, antifouling paints for boats and ships, disinfection of circulating industrial cooling water, and in a slime control in paper factory (3). There is some scientific evidence that TBT is able to masculinize the sex organs of the female of several species of meso- and *neogastropods* resulting in a development of a penis and a vast deference along with the female sex organs in these mollusks (4–6). In addition, it has also been also reported that OTs is able to modulate the immune system behavior in mammalian (2, 7). In an animal model, Ohtaki et al. (8), short-term feeding studies using OTs compounds in rats, observed atrophy of the thymus, decreased numbers of lymphocytes in spleen and lymph nodes, as well as increased serum imunoglobulin M level and decreased serum in imunoglobulin G levels (8). This study also shows that mice exposed to TBT compounds were able to reduce spleen weight and induced the reduction in the number of leukocytes and T helper 2 (Th2) polarization (8). Once, OTs are found in various products, human blood and urine have been used to monitor the human exposure to these compounds. However, there are only a few papers involving these quantifications in human. In 1999, Whalen and colleagues (9) detected OTs in human blood at 64–155 ng/mL levels. This OTs’ presence in human blood was able to compromise the natural killer (NK) cells activity *in vitro* (9). Also, Brown and colleague, in 2017 (10), reported that OTs alters the interleukin 6 (IL-6) secretion from human immune cells and consequently, affect the immune competence (10). In 2014, Valenzuela and colleagues (11) developed an efficient methodology capable to identify 11 OT compounds in urine from harbor workers exposed to antifouling paints. This methodology confirms the possible human contamination by exposure (11).

The complex immune system is able to develop two different kinds of responses, innate and adaptive to fight against foreign pathogens. These two immune responses involve different immunological effector functions; however, these effectors functions work together through compensatory mechanisms with each other to coordinate optimal immune responses (12). Between these two types of immunity response, innate immunity plays important roles in both detecting invading pathogens and developing a specific adaptive immunological response. Therefore, as discussed until now, the adequate innate immune responses are necessary to prevent several infectious diseases (13) and among cells that comprise the immune system, macrophages play a critical role in the innate immune response to pathogens. However, there is no many information about OTs’ (such as TBT) contamination and immune cells in the airway inflammatory disease scenario (Table 1).

**Table 1:** Summary of effects of organotins detected using animal experiment and human cell exposure.

Reference	Human/animal/*vitro*	Experimental designed	Inflammatory mediator	Conclusion
Whalen et al. (9)	Human/*vitro*	Culture	Natural killer cell function	Tributyltin (TBT) affected cell viability
Stridh et al. (43)	Human/*vitro*	Exposed	Lymphocytes apoptosis	TBT induced non-apoptotic morphology
Muller et al. (44)	Human/*vitro*	Exposed	Lymphocytes apoptosis	Lymphocytes more resistant to apoptosis
Ohtaki et al. (8)	C57BL/6 mice	Diet for 2 weeks	Lymphocytes polarization	T helper 2 (Th2) polarization
Kato et al. (2)	C57BL/6 mice	Oral administration of TBT	Modulation of interleukin 10 and IL-12	TBT promotes Th2 polarization
Isomura et al. (18)	*Vitro*	Culture	Neuroblastoma cells (SH-SY5Y)	TBT causes ER stress in human neuroblastoma
Kim et al. (13)	*Vitro*	Culture	Macrophages cells (RAW 264.7 cells)	TBT exerts pathological effects in allergic disease
Kato et al. (19)	C57BL/6 mice	1 µmol/kg TBT (oral gavage)	Modulation of interferon gamma, interleukin 4, and IL-17	TBT induced Treg cells apoptosis
McPherson et al. (14)	Mice (pathogen-free CD-1 male)	i.p. (2.3 mg/kg TMT)	Microglia polarization	TMT elevates M2 anti-inflammatory marker
Brown et al. (10)	Human	Exposed	Interleukin-6	Decrease the secretion of interleukin 6

These cells are sensible to many molecules, including pathogen-associated molecular patterns (PAMPs) and after recognizing by these PAMPs, macrophages are activated, and they induce an inflammatory response by producing pro-inflammatory mediators (13). Kim et al., *in vitro* study, showed that TBT was able to decrease the nitric oxide production in murine macrophage cell line culture (RAW 264.7) and induce significant cell death through mechanisms that varied with the individual chemical (13). The macrophage is an immune cell that is present, as a resident cells, in almost every organ in the body, and they represent the first type of cell that phagocytoses (engulfs a solid particle) foreign materials. Thus, it is possible to assume that most EDCs are phagocytosed by macrophages. However, it is no possible to assume whether these cells are influenced by these chemicals through phagocytosis or through receptor-mediated signaling. McPherson et al., *in vivo study*, reported that the androgen hormone receptor is expressed on macrophages surface and that their signaling through this receptor can modify the function of macrophage (13, 14).

In addition, TBT has been known to reduce the cytotoxic activity of NK cells (15). It has been suggested that many pollutant environmental factors are involved in an aging process, elevation of inflammation and oxidative damage to brain tissue. Reactive oxygen species (ROS), produced from many sources, can react with cellular macromolecules such as proteins, lipids, and DNA. Chemicals products that increase ROS production and inflammation may certainly aggravate the situation and may act as a predisposing factor for neurodegenerative diseases (7, 16). Furthermore, studies have reported that OTs exposure was able to suppress T helper 1 (Th1) response, induce exacerbated Th2 immunity and increase airway inflammation, through a mechanism associated with the apoptosis by T-regulatory cells and increased oxidative stress response (2). In addition, OTs induce macrophage recruitment, leading to necrosis increased, stimulation of an inflammatory cytokines secretion in the local inflammatory site (15). Thus, the main intention of this review is to summarize the late findings involving the inflammatory profile as result of OTs exposure directly and/or indirectly associated with developing the abnormal endocrine function.

## The Role and Mechanism of Th1/Th2 Cells Activation During Inflammatory Response to Respiratory Disease

Tributyltin chloride (TBTC) is an OT compound containing TBT groups that has been used as the heat stabilizer for polyvinyl chloride and catalysts for esterification (3). TBTC is found in industries biocides, wood preservatives, agricultural fungicides, and disinfecting agents in circulating industrial cooling waters, as well as in antifouling paints for marine vessels (3, 17). However, these OTs compounds exhibit various toxicities in mammal organs and organic systems (18) such as adipose tissue, kidney, liver, and lung (19–21), as well as toxicity in the reproductive systems in mammals (3). In pathological features of air pathway inflammatory response, including inflammation induced by the toxicity of TBTC, there is denudation of airway epithelium, collagen deposition, edema, mast cell activation, and inflammatory cell infiltration. Furthermore, the classical inflammatory response induces the elevation of the expression and content of multiple inflammatory mediators in the respiratory tract, including cytokines, chemokines, adhesion molecules, PAMPs, damage-associated molecular pattern, and ROS production (22). This inflammatory scenario results in the activation of immune system and contributes to the recovery of body homeostasis.

The immune system develops both innate (immediate and non-specific) and adaptive (gradual build-up, highly specific, and long-lasting) immune responses to recover the homeostasis in the tissue and also to fight against infection induced by pathogens (23). The first one is orchestrated by neutrophils, monocytes and NK cells that destroy the viruses, bacteria, and fungus, and the second one is orchestrated by lymphocytes (B-cells and T-cells). Lymphocytes B-cells produce antibodies (immunoglobulin), and lymphocytes T-cells are involved primarily in the cell-mediated immune response (23). T-cells and their mediators are involved in inflammation in many diseases scenarios such as diabetes, infectious disease, rheumatoid arthritis, and these cells are likely involved in the pathophysiology of some types of allergy diseases (24). There is two main subpopulations of T-lymphocytes (T-cells). They are differentiated by the presence of cell surface proteins, called cluster of differentiation (CD), and they are classified as lymphocytes CD4 and lymphocytes CD8. T-cell lymphocytes that express CD4 are also known as helper T-cells, and these are regarded as being the most prolific cytokine producers (25).

Cytokines, such as IL-6, tumor necrosis factor alpha (TNF-α), and interferon gamma (IFN-γ), are small proteins that are involved in autocrine, paracrine, and endocrine signaling as immunomodulating agents, and work as the hormonal messengers responsible for most of the biological effects in the immune system, such as cell-mediated immunity and allergic-type responses (25). Thus, activated lymphocytes (T-cell) are important effector cells in the maintenance of health and controlling diseases, such as inflammatory diseases. Lymphocytes T-cells CD4 can be differentiated into two subgroups: lymphocyte helper type 1 (Th1-cell), and lymphocyte helper type 2 (Th2-cell). Th1 and Th2 are distinguished by the types of cytokines they produce, for example: lymphocyte helper type 1 cells (Th1) produce interleukin 2, TNF-α, and IFN-γ; that is, clearly a pro-inflammatory response and lymphocyte helper type 2 cells (Th2) produce interleukin 4 (IL-4), interleukin 10 (IL-10), and interleukin 13 that induces an anti-inflammatory response (23, 26).

There are no many data highlighting the relationship between OTs and activation of lymphocytes T-cells in a Th1 and Th2 response in respiratory diseases. In this sense, most of analyses of the behavior of the immune system in this scenario are in allergic diseases, such as asthma studies. In an allergic inflammation, there is substantial evidence showing a relevant infiltration of lymphocyte helper type 2 cells bronchoalveolar lung tissue. This subpopulation of lymphocytes is increased in the lungs of allergic asthmatics, as well as increased levels of IL-4, interleukin 5, and IL-10 cytokines, and interestingly the level of Th2 cytokines appears to correlate with the severity of disease (23, 27).

Even some authors have shown that polarization of lung lymphocyte profiles clearly correlates with the sequential development of acute allergic (2). Lloyd and Hessel in a nice revision paper, recently published in Nature, discuss findings from many studies. The authors discuss the results of many studies and defend the idea that there are many potential new lymphocytes T-cell lineages, which suggests that the fate of lymphocyte CD4 subsets may be wider than previously thought. Immunological dogma dictates that, following antigen stimulation and several rounds of division, Th1 and Th2 cells become irreversibly committed to these lineages (28). However, the finding that transforming growth factor beta can subvert Th2 cells to the Th9 cell lineage has led to the understanding that effector CD4^+^ T-cell populations might be more plastic than originally thought. In addition, some authors have also shown that Treg lymphocytes and T helper 17 lymphocytes (Th17) cells are not stable populations, and instead have the capacity for dedifferentiation (28). So, the possible therapy in the control of the stimulation of immune system induced by TBT should consider the different types of lymphocytes subpopulations.

## Effects of OT Compounds in Pulmonary System

Organotin compounds show a high toxicity profile in mammals found in reproductive tracts, liver, and immune system (29, 30). In the pulmonary system, although it has been an important route of exposure, the effects of OT compounds are poorly described and have conflicting findings among organisms.

Tributyltin is the most studied group of OT compounds for all toxicological aspects, including its effects on the pulmonary system. Van Loveren et al. (31) showed that rats exposed to a diet containing up to 80 mg/kg of tributyltin oxide (TBTO) have suppressed NK cells activity in the lungs, which have an important role in surveillance, evident against neoplastic and virus-infected targets (31). This potential immunotoxicity in the lungs could favor respiratory viral infections and neoplasms. Contrasting these findings, Carthew et al. (32) examined whether the exposure of rats to TBTO exacerbated the type of pneumonia caused by pneumonia virus of mice and *Mycoplasma* (32). Animals were exposed for 6 weeks to TBTO in the diet, in a similar protocol employed by Van Loveren et al. (31). Although other signs of TBTO toxicity were found, such as a reduction as a reduction in body and thymus weights, as well as the development of cholangitis, there was no evidence that this OTs favored the occurrence of pneumonia in the animals.

Shelton et al. (33) described a case study about a 52-year-old man, who developed asthma after being, exposed to a carpet deodorizer containing TBTO. Acute symptoms such as retrosternal chest pain, nausea and lethargy appeared a few hours of arriving to work and cleared over 2 days at home (33). On returning to work, exacerbations of symptoms (chest tightness and soreness, dry cough, and wheeze) appeared on at least four occasions within 14 weeks. Although this patient had a smoking history, aeroallergens tests, pulmonary function and the temporal relationship with exposure allowed concluding that TBTO was likely the etiologic factor for this patient’s asthma. TBTO also was related to a sore throat, burning nose, and wheezing 24 h after a room had been painted with a paint containing this OT (34). According to Schweinfurth and Gunzel (35), a single 4-h exposure of rats to aerosols of TBTO produced signs of irritation such as nasal discharge, lung edema, and congestion (35).

Although TBT compounds are known to cause irritation of the respiratory tract, eyes, and skin, toxicological data are poorly available. Rats were treated with TBTC, 1 or 5 mg/kg, *via* oral, for 6 weeks. There was observed an increase in lung weight while cell density was reduced 0.3-folds. Indeed, TBTC leads to oxidative stress in the lung, evidenced by ROS production enhancement as well as a bronchi damage with loss of mucosal epithelial lining and fibrocartilaginous shell (36). In a man, cough and difficulty in breathing, characterized by inspiratory discomfort, were observed a few hours after inhaling an unspecified amount of powdered TBTC (37). Shortness of breath and chest discomfort was still present 20 days after the exposure.

In addition, OTs are capable of producing changes in the respiratory system of different organisms. Exposure of man and animals to tricyclohexyltin compounds (tricyclohexyltin hydroxide and tricyclohexyltriazolyltin) employed in agriculture as acaricides lead to severe irritation of airways and pulmonary tissue. In animals, these compounds caused pulmonary lesions and worsening to lung edema after oral or intravenous administration (38).

Triphenyltin (TPT) administered intraperitoneally in rabbits at 16 mg/kg (LD_50_) immediately lead to hyperpnea. However, beagle dogs dosed with up to 0.62 mg/kg/day of TPT for up to 52 weeks did not show any gross or microscopic alterations in the respiratory tract (39). According to Olushola Sunday et al. (30), tetraorganotins compounds produce respiratory failure as acute effects in mice and dogs, similar to those seen in triorganotins poisoning (30). In a man, cough and difficulty in breathing, characterized by inspiratory discomfort, were observed a few hours after inhaling an unspecified amount of powdered trimethyltin chloride (37). Shortness of breath and chest discomfort was still present 20 days after the exposure.

Diethyltin dichloride produces nasal irritation in mammals after topical administration (40). In rabbits, this compound increased the respiratory rate at lower doses (3–5 mg/kg) (41). Diphenyltin caused generalized weakness and difficulty with respiration in rats that received 100 mg/kg intraperitoneally (42).

## Conclusion

Although the OTs are widely used in the agro-industry with extreme contact for both human respiratory tracts and skin, the consequence of this exposure remains poorly investigated. Furthermore, the most recent data involving airway inflammation by OTs are from the early 2000s, nearly 20 years ago (2). Considering the context of exposure to stannous compounds, the respiratory system is considered one of the main forms of contact with these toxicants, reinforcing the importance of establishing—or not—a causal relationship between exposure and respiratory diseases.

## Author Contributions

AN-S, DD, HS, RF, KF, and CC helped to draft the manuscript. LR and IS participated in the study’s design. JG and LL contributed to the conception, design, and supervision of the study. LMA answered the reviewer’s comments point by point and reviewed all manuscript before submission the final version.

## Conflict of Interest Statement

The author, CC, was employed by a company called Cooperativa de Consumo dos Servidores do DER/MG (COOPEDER). This commercial company has a partnership with the Laboratório de Obesidade e Reprodução at the Federal University of Espirito Santo. Thus, none of the authors of this manuscript has a financial or personal relationship with other people or organizations that could inappropriately influence or bias the content of the manuscript.
